# Supplementary material for: Pyruvate Dehydrogenase Kinase 1 inhibition mediated oxidative phosphorylation enhancement in cartilage promotes osteoarthritis progression
Source: BMC Musculoskelet Disord. 2023 Jul 20;24:597. doi: 10.1186/s12891-023-06585-6 (PMC10357736; doi:10.1186/s12891-023-06585-6)
Supplement: Supplementary file 1 — Additional file 1. [file 12891_2023_6585_MOESM1_ESM.zip › KEGG.Gsea.1653622432464/KEGG_AUTOIMMUNE_THYROID_DISEASE.html]

Details for gene set KEGG\_AUTOIMMUNE\_THYROID\_DISEASE[GSEA]

|  || Dataset | 55457\_collapsed\_to\_symbols.cls.cls #OA\_versus\_normal.cls.cls #OA\_versus\_normal\_repos |
| Phenotype | cls.cls#OA\_versus\_normal\_repos |
| Upregulated in class | normal |
| GeneSet | KEGG\_AUTOIMMUNE\_THYROID\_DISEASE |
| Enrichment Score (ES) | -0.2055672 |
| Normalized Enrichment Score (NES) | -0.76617265 |
| Nominal p-value | 0.85858583 |
| FDR q-value | 0.9663527 |
| FWER p-Value | 1.0 |
Table: GSEA Results Summary

  

Fig 1: Enrichment plot: KEGG\_AUTOIMMUNE\_THYROID\_DISEASE      
 Profile of the Running ES Score & Positions of GeneSet Members on the Rank Ordered List

  

| SYMBOL | TITLE | RANK IN GENE LIST | RANK METRIC SCORE | RUNNING ES | CORE ENRICHMENT || 1 | HLA-DMB | "major histocompatibility complex, class II, DM beta [Source:HGNC Symbol;Acc:HGNC:4935]" | 679 | 0.598 | 0.0079 | No |
| 2 | CD86 | CD86 molecule [Source:HGNC Symbol;Acc:HGNC:1705] | 689 | 0.596 | 0.0678 | No |
| 3 | IFNA8 | interferon alpha 8 [Source:HGNC Symbol;Acc:HGNC:5429] | 1206 | 0.475 | 0.0758 | No |
| 4 | HLA-DQA1 | "major histocompatibility complex, class II, DQ alpha 1 [Source:HGNC Symbol;Acc:HGNC:4942]" | 1334 | 0.452 | 0.1119 | No |
| 5 | HLA-DMA | "major histocompatibility complex, class II, DM alpha [Source:HGNC Symbol;Acc:HGNC:4934]" | 1769 | 0.388 | 0.1176 | No |
| 6 | HLA-DOB | "major histocompatibility complex, class II, DO beta [Source:HGNC Symbol;Acc:HGNC:4937]" | 2147 | 0.344 | 0.1232 | No |
| 7 | HLA-DQB1 | "major histocompatibility complex, class II, DQ beta 1 [Source:HGNC Symbol;Acc:HGNC:4944]" | 2840 | 0.266 | 0.0964 | No |
| 8 | FAS | Fas cell surface death receptor [Source:HGNC Symbol;Acc:HGNC:11920] | 3828 | 0.173 | 0.0373 | No |
| 9 | FASLG | Fas ligand [Source:HGNC Symbol;Acc:HGNC:11936] | 3931 | 0.162 | 0.0458 | No |
| 10 | IFNA4 | interferon alpha 4 [Source:HGNC Symbol;Acc:HGNC:5425] | 4282 | 0.129 | 0.0317 | No |
| 11 | HLA-DOA | "major histocompatibility complex, class II, DO alpha [Source:HGNC Symbol;Acc:HGNC:4936]" | 4344 | 0.125 | 0.0396 | No |
| 12 | CD28 | CD28 molecule [Source:HGNC Symbol;Acc:HGNC:1653] | 4543 | 0.110 | 0.0353 | No |
| 13 | TSHR | thyroid stimulating hormone receptor [Source:HGNC Symbol;Acc:HGNC:12373] | 5112 | 0.063 | -0.0025 | No |
| 14 | IL5 | interleukin 5 [Source:HGNC Symbol;Acc:HGNC:6016] | 5277 | 0.050 | -0.0102 | No |
| 15 | HLA-DPB1 | "major histocompatibility complex, class II, DP beta 1 [Source:HGNC Symbol;Acc:HGNC:4940]" | 5486 | 0.033 | -0.0229 | No |
| 16 | CD40LG | CD40 ligand [Source:HGNC Symbol;Acc:HGNC:11935] | 5529 | 0.030 | -0.0231 | No |
| 17 | IL2 | interleukin 2 [Source:HGNC Symbol;Acc:HGNC:6001] | 5819 | 0.007 | -0.0449 | No |
| 18 | IFNA2 | interferon alpha 2 [Source:HGNC Symbol;Acc:HGNC:5423] | 5869 | 0.004 | -0.0483 | No |
| 19 | IFNA21 | interferon alpha 21 [Source:HGNC Symbol;Acc:HGNC:5424] | 6127 | -0.015 | -0.0668 | No |
| 20 | GZMB | granzyme B [Source:HGNC Symbol;Acc:HGNC:4709] | 6132 | -0.015 | -0.0656 | No |
| 21 | TPO | thyroid peroxidase [Source:HGNC Symbol;Acc:HGNC:12015] | 6467 | -0.040 | -0.0875 | No |
| 22 | HLA-DRA | "major histocompatibility complex, class II, DR alpha [Source:HGNC Symbol;Acc:HGNC:4947]" | 6550 | -0.046 | -0.0893 | No |
| 23 | IL4 | interleukin 4 [Source:HGNC Symbol;Acc:HGNC:6014] | 6673 | -0.054 | -0.0933 | No |
| 24 | IFNA10 | interferon alpha 10 [Source:HGNC Symbol;Acc:HGNC:5418] | 6682 | -0.054 | -0.0884 | No |
| 25 | IFNA5 | interferon alpha 5 [Source:HGNC Symbol;Acc:HGNC:5426] | 6755 | -0.059 | -0.0879 | No |
| 26 | CD40 | CD40 molecule [Source:HGNC Symbol;Acc:HGNC:11919] | 6825 | -0.065 | -0.0867 | No |
| 27 | CD80 | CD80 molecule [Source:HGNC Symbol;Acc:HGNC:1700] | 6918 | -0.074 | -0.0864 | No |
| 28 | HLA-DRB1 | "major histocompatibility complex, class II, DR beta 1 [Source:HGNC Symbol;Acc:HGNC:4948]" | 7087 | -0.086 | -0.0907 | No |
| 29 | HLA-F | "major histocompatibility complex, class I, F [Source:HGNC Symbol;Acc:HGNC:4963]" | 7196 | -0.096 | -0.0894 | No |
| 30 | TG | thyroglobulin [Source:HGNC Symbol;Acc:HGNC:11764] | 7217 | -0.097 | -0.0811 | No |
| 31 | IFNA6 | interferon alpha 6 [Source:HGNC Symbol;Acc:HGNC:5427] | 7272 | -0.101 | -0.0751 | No |
| 32 | TSHB | thyroid stimulating hormone subunit beta [Source:HGNC Symbol;Acc:HGNC:12372] | 7342 | -0.105 | -0.0697 | No |
| 33 | PRF1 | perforin 1 [Source:HGNC Symbol;Acc:HGNC:9360] | 8265 | -0.176 | -0.1235 | No |
| 34 | IFNA7 | interferon alpha 7 [Source:HGNC Symbol;Acc:HGNC:5428] | 8533 | -0.196 | -0.1244 | No |
| 35 | CGA | "glycoprotein hormones, alpha polypeptide [Source:HGNC Symbol;Acc:HGNC:1885]" | 8583 | -0.200 | -0.1079 | No |
| 36 | IFNA1 | interferon alpha 1 [Source:HGNC Symbol;Acc:HGNC:5417] | 8640 | -0.204 | -0.0916 | No |
| 37 | IL10 | interleukin 10 [Source:HGNC Symbol;Acc:HGNC:5962] | 8855 | -0.221 | -0.0857 | No |
| 38 | HLA-DPA1 | "major histocompatibility complex, class II, DP alpha 1 [Source:HGNC Symbol;Acc:HGNC:4938]" | 9481 | -0.273 | -0.1067 | No |
| 39 | CTLA4 | cytotoxic T-lymphocyte associated protein 4 [Source:HGNC Symbol;Acc:HGNC:2505] | 10739 | -0.398 | -0.1640 | Yes |
| 40 | HLA-G | "major histocompatibility complex, class I, G [Source:HGNC Symbol;Acc:HGNC:4964]" | 10869 | -0.413 | -0.1322 | Yes |
| 41 | HLA-E | "major histocompatibility complex, class I, E [Source:HGNC Symbol;Acc:HGNC:4962]" | 11659 | -0.526 | -0.1401 | Yes |
| 42 | HLA-A | "major histocompatibility complex, class I, A [Source:HGNC Symbol;Acc:HGNC:4931]" | 12502 | -0.749 | -0.1295 | Yes |
| 43 | HLA-B | "major histocompatibility complex, class I, B [Source:HGNC Symbol;Acc:HGNC:4932]" | 12534 | -0.764 | -0.0543 | Yes |
| 44 | HLA-DRB4 | "major histocompatibility complex, class II, DR beta 4 [Source:HGNC Symbol;Acc:HGNC:4952]" | 12636 | -0.815 | 0.0205 | Yes |
Table: GSEA details [plain text format]

  

Fig 2: KEGG\_AUTOIMMUNE\_THYROID\_DISEASE      
 Blue-Pink O' Gram in the Space of the Analyzed GeneSet

  

Fig 3: KEGG\_AUTOIMMUNE\_THYROID\_DISEASE: Random ES distribution      
 Gene set null distribution of ES for **KEGG\_AUTOIMMUNE\_THYROID\_DISEASE**
